# Electroencephalogram-Based Facial Gesture Recognition Using Self-Organizing Map

**DOI:** 10.3390/s24092741

**Published:** 2024-04-25

**Authors:** Takahiro Kawaguchi, Koki Ono, Hiroomi Hikawa

**Affiliations:** Faculty of Engineering Science, Kansai University, Osaka 564-8680, Japan

**Keywords:** self-organizing map, EEG, facial gesture

## Abstract

Brain–computer interfaces (BCIs) allow information to be transmitted directly from the human brain to a computer, enhancing the ability of human brain activity to interact with the environment. In particular, BCI-based control systems are highly desirable because they can control equipment used by people with disabilities, such as wheelchairs and prosthetic legs. BCIs make use of electroencephalograms (EEGs) to decode the human brain’s status. This paper presents an EEG-based facial gesture recognition method based on a self-organizing map (SOM). The proposed facial gesture recognition uses α, β, and θ power bands of the EEG signals as the features of the gesture. The SOM-Hebb classifier is utilized to classify the feature vectors. We utilized the proposed method to develop an online facial gesture recognition system. The facial gestures were defined by combining facial movements that are easy to detect in EEG signals. The recognition accuracy of the system was examined through experiments. The recognition accuracy of the system ranged from 76.90% to 97.57% depending on the number of gestures recognized. The lowest accuracy (76.90%) occurred when recognizing seven gestures, though this is still quite accurate when compared to other EEG-based recognition systems. The implemented online recognition system was developed using MATLAB, and the system took 5.7 s to complete the recognition flow.

## 1. Introduction

Brain–computer interface (BCI) technology allows information to be sent directly from the human brain to a computer. BCIs make use of electroencephalogram (EEG) or electrocortical wave (ECoG) signals to decode the status of the human brain. In contrast to the EEG, which records signals generated by nerve electrical activity in a non-invasive manner, ECoG measurement is invasive and requires clinical surgery to place electrodes on the surface of the brain. Deciphering and recognizing these signals is an essential technology for the practical application of BCIs.

BCIs enhance the ability of human brain activities to interact with the environment, and they have been used in the context of game interaction, robot control, emotion recognition, fatigue detection, sleep quality assessment, and clinical fields such as abnormal brain disease detection and prediction, including applications to seizures, Parkinson’s disease, Alzheimer’s disease, and schizophrenia [1]. Changes in emotions cause differences in the EEG signals, and EEG-based emotion recognition has been widely utilized in human–computer interaction, medical diagnosis, the military, and other fields [2]. The most desired applications for BCIs include EEG-based brain-controlled mobile robots and robotic limbs, which can be a powerful aid in helping people with disabilities move voluntarily [3]. Another interesting BCI application area is real-time remote control over unmanned aerial vehicles (UAVs) [4], and Lin et al. [5] proposed decoding facial gestures using EEG signals to control the UAV.

In the EEG signal analysis, it is necessary to reduce the influence of contamination caused by muscle activities such as eye movements, but this is a very challenging problem. One of the popular methods is artifact subspace reconstruction (ASR), which is an automatic component-based mechanism to effectually remove large-amplitude or transient artifacts that contaminate EEG data. Then, the cleaned EEG signal is analyzed by a classifier to estimate the brain status. Recent advances in machine learning algorithms and artificial neural networks (ANNs) have spawned increased interest and research in EEG-based BCI applications since they can be used as the classifier. State-of-the-art machine learning and deep learning algorithms have been utilized in a variety of BCI applications to detect, monitor, and maintain human cognitive state and task performance [1]. The self-organizing map (SOM) [6] is a special type of ANN that performs nonlinear mapping from a given high-dimensional input vector space to a lower-dimensional map of neurons. It has been utilized to visualize, interpret, and classify high-dimensional vectors in applications such as magnetic resonance image (MRI) segmentation [7], the tracking of objects in video sequences [8], cluster visualization [9], and video highlight generation [10]. In our previous work [11], we proposed the SOM-Hebb classifier and applied it to a hardware hand sign recognition system. The SOM-Hebb classifier is a hybrid network consisting of an SOM and a single-layer feedforward neural network. The SOM in the classifier performs clustering of the hand sign data, and the feedforward neural network labels the clusters by using a simple Hebbian learning algorithm. In relation to the brain, Kohonen [12] demonstrated that an SOM has a self-organizing ability similar to that of a biological brain.

In this paper, we present a facial gesture recognition system using the SOM-Hebb classifier to identify facial gestures from EEG signals. Our main contributions are as follows.

- Muscle activity such as eye movement is known to influence EEG analysis, so these artifact signals are typically removed during preprocessing to minimize contamination of the EEG signal [1]. These signals are presumably better able to represent changes in facial expressions. To effectively detect facial gestures, the proposed system utilizes these artifact signals without filtering.
- We demonstrate the use of the SOM-Hebb classifier in the field of EEG-based recognition.
- We present an online recognition system developed using the proposed face gesture recognition. Many researchers use EEG or ECoG datasets to evaluate their methods, but we experimentally verified the recognition performance of our proposed method in near real-time using the online system.

The remainder of this paper is organized as follows. Section 2 of this paper reviews various BCI systems in the literature. The recognition algorithm including the SOM-Hebb classifier is presented in Section 3. The definition of facial gestures, the online experiment system, and the experimental results are discussed in Section 4. We conclude in Section 5 with a brief summary and mention of future work.

## 2. Related Work

Brain measurement techniques are broadly divided into intracranial invasive types and non-invasive types. The non-invasive techniques include EEG measurement and nuclear magnetic resonance imaging (fMRI). The invasive techniques such as ECoG involve a surgical procedure that places electrodes on the surface of the brain.

Accurately decoding brain states requires the extraction of valid features from raw EEG or ECoG signals. Regarding feature selection, Wang et al. [13] proposed a feature reduction method based on the Pearson correlation coefficient and applied it to their hand gesture recognition system that utilizes EEG signals. Their feature reduction method can effectively reduce the feature extraction time and improve the classification accuracy.

Mohseni et al. [14] proposed a motor imagery hand gesture decoding method that predicted intended hand grasps from EEG data. For motor imagery hand gestures, EEG signals are recorded while subjects are imagining the gesture. Recognizing motor imagery gestures is more difficult than recognizing motor execution gestures, so the authors proposed a hierarchical common spatial pattern (HCSP) algorithm to predict intended hand grasps from EEG data. Experimental results showed that the classification accuracy is more than five times higher than the level of chance.

The influence of EEG electrode density on gesture decoding accuracy was investigated by Schreiner et al. [15]. For hand gesture recognition, the ultra-high-density EEG (uHD EEG) was recorded using a total of 352 and 256 electrodes placed over the sensorimotor cortex. Experimental results showed that classification models based on the conventional EEG performed worse than the uHD EEG-based system.

Gesture classification is typically performed by a classifier using information contained in EEG or ECoG signals. Zhang et al. [16] proposed a novel hand movement recognition system with a long short-term memory (LSTM) network featuring an attention mechanism to learn the electroencephalogram (EEG) time-series information. Extensive experiments on the EEG Movement Dataset [17] showed that this method outperformed several state-of-the-art methods in both intra-subject and cross-subject validation schemes. In the within-subject scheme, the system is trained on a target user and utilized for the BCI applications on the same user. As for the cross-subject scheme, it provides a generalized solution that performs across subjects once trained on a dataset.

Various classifiers have been utilized to classify EEG signals. Shilaskar et al. [18] compared four classifiers—random forest, decision tree, Adaboost, and SVM—by testing them on two gesture recognition problems and found that the random forest classifier achieved the highest accuracy (78.62%), followed by the decision tree and Adaboost.

ECoG measurements can record brain signals with higher temporal and spatial resolution compared to non-invasive measurements. The ECoG signals provide information up to 500 Hz, which covers the so-called high-gamma activation (HGA). Since the ECoG electrodes are small and closely spaced, they provide much higher spatial resolution than the EEG, allowing for more precise mapping of functional brain regions. Therefore, invasive ECoG measurement is advantageous in terms of measuring high-quality brain activity.

The hand posture detection method proposed by Kapeller et al. [19] utilizes ECoG signals to detect individual finger movements. This method reduced the detection error from 16.16% to 4.20% by selecting features from the ECoG signal using the common spatial pattern (CSP) algorithm.

Pradeepkumar et al. [20] proposed a hand gesture recognition system utilizing ECoG signals and tested the recognition performance using the finger flex dataset [21], which contains ECoG recordings of three anonymous patients with corresponding finger movement information. Using feature reduction based on statistical analysis and an LSTM neural network, their system achieved a classification accuracy of 82.4%.

Liao et al. [22] applied both EEG and ECoG signals to decode finger movements and then compared classification accuracy utilizing an SVM classifier. The decoding accuracy using an EEG was 77.11% and using ECoG was 91.28%. These results indicate that ECoG signals are suitable for measuring brain function. However, the ECoG method requires invasive measurements as well as surgery to place electrodes directly on the surface of brain tissue, making it difficult to apply in BCIs.

Regarding the use of an SOM in BCIs, Wang et al. [23] utilized an SOM to detect distracted driving, which is a significant cause of traffic accidents, from EEG signals. The accuracy of their system approached approximately 90% for the recognition of distracted and concentrated driving according to the selected frontal and left motor components. Bueno et al. [24] proposed another SOM-based system that classifies user intention from the EEG signal. In their experiment, three tasks were recognized: evoking words that start with the same letter, imagining self-paced movements of the left hand, and imagining self-paced movements of the right hand. Utilizing frequency features, their system successfully identified three different brain tasks with an accuracy of 71.21%.

## 3. Materials and Methods

**Figure 1:**
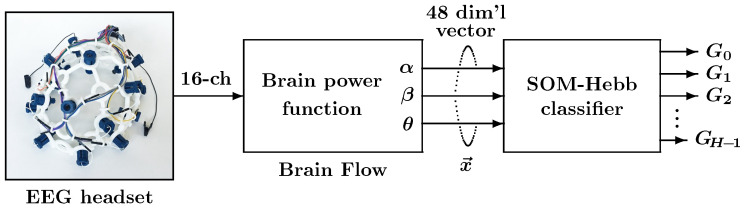
Configuration of the proposed facial recognition system. The system consists of an EEG headset, Brain Flow, and SOM-Hebb classifier.

Figure 1 shows the data flow of the proposed facial gesture recognition method, which consists of an EEG headset, feature extractor, and SOM-Hebb classifier.

### 3.1. EEG Signal Acquisition

The EEG headset reads brain signals that represent human brain activity. We utilized the Ultracortex Mark IV EEG headset with OpenBCI, an open-source brain–computer interface. The headset was equipped with 16 electrons spread over the surface of the human head. The 16 electrodes acquire EEG signals, which are sampled at the rate of $f_{S}=125$ Hz to be sent to a computer via Bluetooth. The computer then performs facial gesture recognition.

### 3.2. Feature Extraction

The range of frequencies observed in a healthy human EEG are between 1 and 30 Hz, and amplitudes will vary between 20 and 100 μV. In the proposed system, subdivided band power features for the α (8–13 Hz), β (14–30 Hz), and θ (4–7 Hz) signals were exploited for gesture recognition. The α signal appears during relaxed state, and the β signal is generated during the awake, focusing, or tense state. The θ signal is another EEG signal, which occurs during sleep or meditative state. This research aims to develop a system that performs online recognition; thus, the EEG signal measurement must be quickly performed. The δ EEG signal was not used because its frequency is lower than that of the θ signal and it would take more time to measure accurately. To extract these band power features, we utilized the brain power function included in Brain Flow, which is a commonly used library to analyze data from biosensors such as the EEG headset. The brain power function generates α(ch), β(ch), and θ(ch), where ch is the electrode channel number (ch=1,2,⋯16).

Then, α(ch), β(ch), and β(ch) are merged into a vector $\vec{x}$.

(1) $$\vec{x}=\left\{\xi_{0},\xi_{1},\cdots ,\xi_{D-1}\right\}\in {ℜ}^{D}.$$

Since we used an EEG headset with 16 electrodes channels, the dimensions of $\vec{x}$ are D=48.

### 3.3. SOM-Hebb Classifier

**Figure 2:**
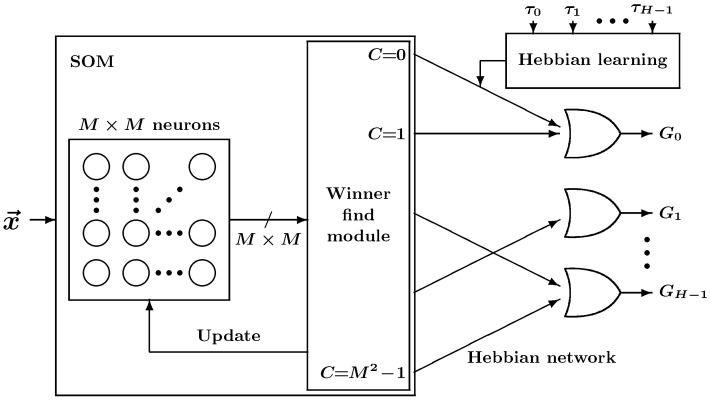
SOM-Hebb classifier comprising an SOM with M×M neurons and a Hebbian learning network.

From $\vec{x}$, facial gestures are identified by the SOM-Hebb classifier, as shown in Figure 2. This SOM-Hebb classifier, which is the same one used in our previous work [11], is a hybrid network consisting of an SOM and a single-layer feedforward neural network trained with the generalized Hebbian algorithm. The classifier reads the *D*-dimensional vectors from the brain power function and classifies them into *H* classes.

An SOM is an unsupervised neural network machine learning method consisting of M×M neurons. Each neuron contains a *D*-dimensional weight vector. The weight vector of neuron *k* is defined as

(2) $$\vec{m_{k}}=\left\{\mu_{k,0},\mu_{k,1},\cdots ,\mu_{k,D-1}\right\}\in {ℜ}^{D}.$$

The operation of an SOM is divided into two phases: learning and recall. It is trained using a set of input vectors during the learning phase, which establishes the weight map. During the learning phase, vectors $\vec{x}$ in (1) are given to the SOM in multiple iterations, and the weight vectors are adjusted to represent the input vector space. For each input vector, distances to all weight vectors are calculated. Euclidean distance is utilized to represent the vector distance.

(3) $$d_{k}=\sqrt{\sum_{j=0}^{D-1}{\left(\xi_{j}-\mu_{kj}\right)}^{2}}$$

The winner neuron *C* is then determined as the one having the weight vector closest to the input vector.

(4) $$C=\arg\min_{k}d_{k}.$$

After the winner neuron is determined, the vectors of this neuron and its neighborhood neurons are updated so that they are closer to the input vector. The weight vector update is given by following equation.

(5) $$\vec{m_{k}}\left(t+1\right)=\vec{m_{k}}\left(t\right)+h_{Ck}\cdot \left\{\vec{x}-\vec{m_{k}}\left(t\right)\right\}$$

$h_{Ck}$ determines the amount of the weight update and is called the neighborhood function. It is calculated by

(6) $$h_{Ck}=\alpha \left(t\right)\exp\left(-\frac{\|\vec{r_{C}}-\vec{r_{k}}\|}{2\sigma^{2}\left(t\right)}\right),$$

where $\vec{r_{C}}\in {ℜ}^{2}$ and $\vec{r_{i}}\in {ℜ}^{2}$ are the location vectors of the winner neuron *C* and neuron *k*, respectively. The neighborhood function provides a topology-preserving nature, i.e., two vectors that are neighbors in the input space will also be represented close to each other on the map.

In the recall phase, only the winner search is carried out, while the trained weight vectors are retained. Every gesture makes one of the neurons a winner, and the class of the gesture is identified from the winner neuron number *C*. In this process, each neuron must be associated with one of the gesture classes, which is referred to as labeling. This class association is carried out using Hebbian learning, which is supervised learning.

Training vectors and their teaching data are sequentially fed to the network during the Hebbian learning phase. The teaching data, $\tau_{0},\tau_{1},\cdots \tau_{H-1}$, indicate the class of the training vectors. Then, the winner neuron is associated with the corresponding class $\tau_{l}$ if a strong correlation is found between them. Specifically, for each neuron, the number of times the neuron wins and the class that caused that win are recorded during training. The neuron is then associated with the class that made that neuron win more than other classes. Some neurons may not become winners, and those neurons are not associated with any gesture class. False recognition occurs when one such neuron is chosen as the winner during the recognition phase. Such neurons are disabled after training to avoid false recognition, and the disabled neurons are excluded from the winner search.

## 4. Results

### 4.1. Facial Gestures

It is well known that muscle contraction and stretch near the recording site generate artifacts in EEG signals [1]. Specifically, when recording the EEG signals, the artifacts are generated by facial muscle movements, such as eye blinks or eye movements. The degree of the muscle contraction and stretch affect the amplitude and waveform of artifacts in EEG signals. When analyzing pure brain activity, such signal changes are considered to contaminate the EEG signal and are therefore removed. The unique point of the proposed method is that it actually utilizes these EEG signal artifacts and defines gestures by combining facial movements that have a large influence, thereby achieving high recognition accuracy.

**Figure 3:**
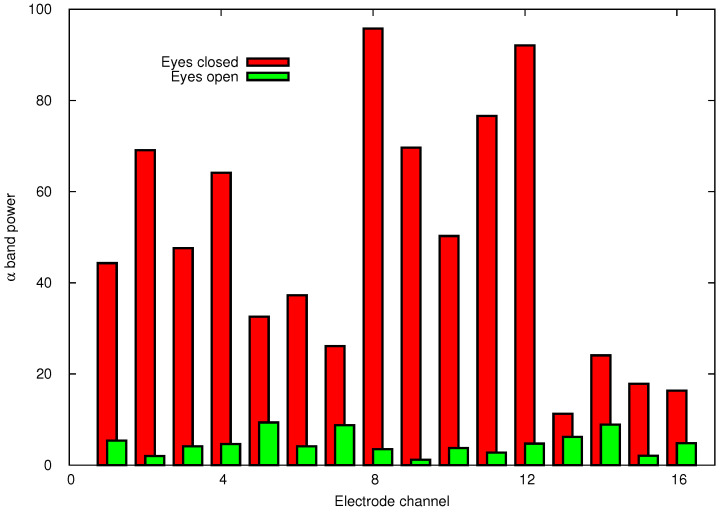
α band strength for eyes closed and open.

**Figure 4:**
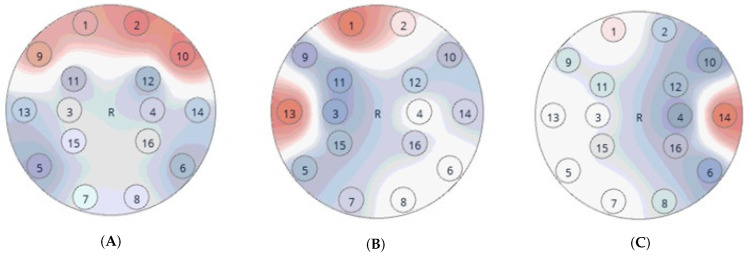
Head plots for (**A**) eyes wide open, (**B**) left teeth clenched, and (**C**) right teeth clenched. The deeper the red in a region, the more brain activity is occurring.

To define facial gestures, the influence of muscle movements was first measured. Figure 3 and Figure 4 show facial movements that had a major impact on the brain wave sinuses. Figure 3 depicts the α band signal strength of 16 channels when the eyes are open (green bars) and closed (red). As shown, the signal level when opening and closing the eyes clearly affects the alpha signal, which is significantly higher when the eyes are closed. Figure 4 shows head plots. These head plots were measured using the OpenBCI GUI software v5.2.2 (access date: 21 August 2023, https:docs.openbci.com). The head plot displays which regions of the head are experiencing the most activity. The deeper the red in a region, the more brain activity is occurring in that region. In Figure 4A the electrodes placed in front of the head receive higher level signals if the eyes are open even wider. We found that clenching teeth also affects the EEG signals. Figure 4B,C show the signal intensities of the 16 electrodes when the left and right teeth are clenched. In Figure 4B, the left electrode (no. 13) is receiving a strong signal when the left teeth are clenched. Similarly, in Figure 4C the signal received by the right electrode (no. 14) is stronger than that of the other electrodes when the right teeth are clenched.

On the basis of the above observations, we define the following seven facial gestures.

**G1:**  Open eyes;
**G2:**  Close eyes;
**G3:**  Open eyes and clench right teeth;
**G4:**  Open eyes and clench left teeth;
**G5:**  Open eyes wide;
**G6:**  Close eyes and clench right teeth;
**G7:**  Close eyes and clench left teeth.

### 4.2. Experimental System

Utilizing the facial gesture recognition algorithm discussed in the previous section, we developed an online facial gesture recognition system using MATLAB (24.1.0.2508561 (R2024a)). During the recognition, the SOM performs the winner search only, no weight adjustment is performed, and offline learning for the SOM-Hebb classifier is carried out beforehand, where the SOM-Hebb classifier is trained using the training dataset.

#### 4.2.1. Offline Learning

**Figure 5:**
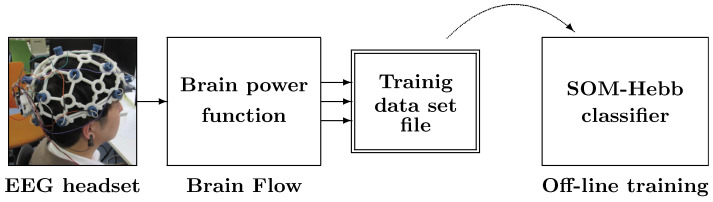
Acquisition of training data and offline training of SOM-Hebb classifier.

Training of the SOM-Hebb classifier was carried out by offline training as shown in Figure 5. First, the training dataset for the offline learning was acquired from a single user. Each gesture signal was recorded for 2 s. This was repeated 30 times, and the recorded signals were converted to $\vec{x}$ vectors. The measurement time and the number of learning data were mainly determined so as not to burden the EEG measurement subjects. As explained earlier, the lowest frequency of the band power feature used is 4 Hz (θ). The recording time was set to 2 s in order to measure a sufficient number of cycles included in the feature signal without imposing too much physical or mental stress on the subject. The number of learning vectors was set to 30, simply taking into account the degree of fatigue of the subjects during the EEG measurements. Therefore, the number of training vectors for each gesture is 30, and the training dataset consists of 210 vectors in total.

The implemented system comprises an SOM made of 8 × 8 neurons. During offline learning, all weight vectors of the neurons are determined as well as the connections between neurons and gesture classes as discussed in the previous section. These trained weight vectors and connections are then utilized in the online recognition for the facial gestures.

In order to investigate the relationship between the number of classes and the recognition rate, the SOM-Hebb classifier was trained using different numbers of gesture classes. We conducted training using the following four sets of classes.

- Two-gesture recognition: (G1, G2)
- Four-gesture recognition: (G1, G2, G3, G4)
- Five-gesture recognition: (G1, G2, G3, G4, G5)
- Seven-gesture recognition: (G1, G2, G3, G4, G5, G6, G7)

#### 4.2.2. Online Facial Gesture Recognition System

**Figure 6:**
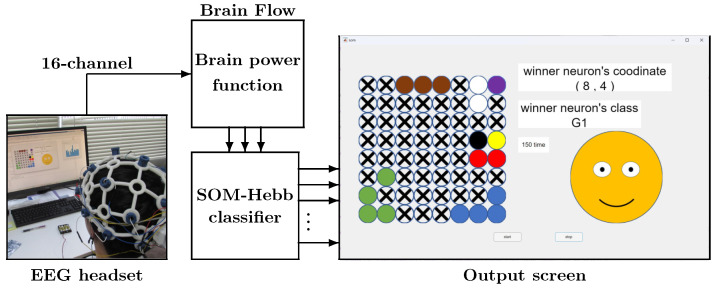
Online facial gesture recognition system. Colored circles are the neurons associated with gesture classes (black: G1, white: G2, blue: G3, Red: G4, green: G5, purple: G6, blown: G7, yellow: Winner neuron).

Figure 6 shows the online facial gesture recognition system. The EEG signals when the user is performing a facial gesture are captured online over the period of two seconds. Then, they are immediately converted into vector $\vec{x}$, and the SOM-Hebb classifier predicts the gesture class. The SOM-Hebb classifier utilizes weight vectors and neuron–class connections that are result of the offline training discussed in the previous section. The predicted gesture class given by the SOM-Hebb classifier is displayed on the output screen, along with other information. Circles placed in an 8 × 8 grid on the output screen are neurons, and colored circles are the neurons associated with gesture classes. The output display in Figure 6 is for seven gestures, and the color indicates the gesture class associated with each neuron.

**Figure 7:**
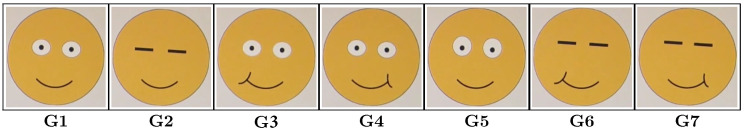
Illustrations indicating gestures.

Note that the neuron colored in yellow is the winning neuron. Figure 6 shows that the neuron placed at (8, 4) is the winner, and the recognition result is G1 since the neuron is associated with G1. In this example, multiple neurons are associated with a single class. This is because the winning neurons were associated with the same gesture class signals during the offline learning. Due to the topology-preserving nature of an SOM, these neurons are also close to each other on the map. Circles with a cross are the disabled neurons, which do not become winners. The right side of the screen displays the coordinates of the winning neuron, the predicted gesture class, and an illustration of that gesture. The illustrations indicating the facial gestures are summarized in Figure 7.

### 4.3. Facial Gesture Recognition

The recognition performance of the proposed system was examined by a single user (a video of the experiment is available at http://www2.itc.kansai-u.ac.jp/~hikawa/BCI/facegesture.mp4, access date: 14 April 2023).

Four recognition tests were conducted using different numbers of gesture classes. The user was asked to sit on a chair and was instructed to perform one of the facial gestures. The predicted and actual gesture classes were then recorded. In the experiments, each gesture was performed 30 times, except for in the two-gesture experiment, where each gesture was performed 50 times.

**Figure 8:**
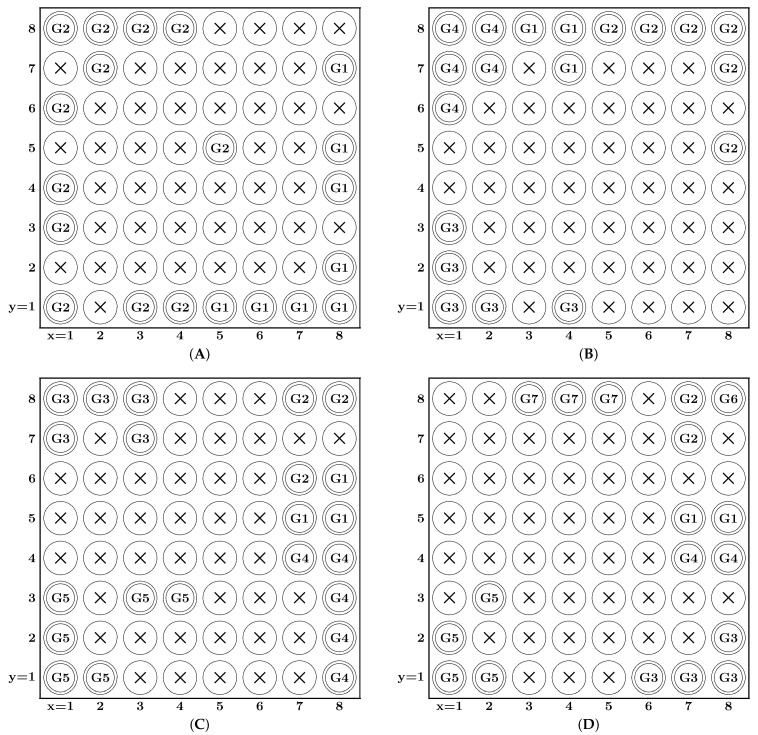
Neuron map of the SOM trained for (**A**) two facial gestures, (**B**) four facial gestures, (**C**) five facial gestures, (**D**) seven facial gestures.

The recognition experiment described above was conducted twice on different days. The results of the recognition experiments are summarized in Table 1. Note that the results of both experiments show similar recognition accuracy. The recognition accuracy for the number of gestures recognized by the proposed system is high at 97.57%, 94.35%, 92.67%, and 76.90%, but the accuracy clearly decreases as the number of gesture classes increases. In order to find the cause of the degradation in recognition rate, we conducted a more detailed verification. Table 2, Table 3, Table 4 and Table 5 show the confusion lists of experiment 1 with two, four, five, and seven types of gestures. Figure 8 shows the neuron maps that were used for the two-, four-, five-, and seven-class facial gesture recognition. Each map shows neurons for facial gesture labels assigned to neurons in the SOM, which were determined by the Hebbian learning. Circles with a cross are disabled neurons, which were not selected as winners.

Table 5 shows that the recognition accuracy for gestures G5 (eyes wide open) and G6 (closed eyes and clenched right teeth) is significantly lower than the other gestures. As shown in Figure 3, when the eyes are wide open, the signal level of the front electrodes becomes high. Clenching the right teeth (G3) and left teeth (G4) increases the signal level at electrodes 14 and 13, respectively. As shown in Figure 4, electrodes 13 and 14 are near the region affected by G5, which is presumably why G5 was misrecognized as G3 or G4. Table 4 shows that several G5 gestures were incorrectly recognized as G4, which could be for similar reasons. Gesture G6 was often confused with G3, but the difference between them is whether the eyes are open or closed. Figure 8D shows that gesture G6 was assigned to only one neuron, which is far fewer than the other gestures. The reason just one neuron was assigned to G6 can be traced to the small variation in the training data of G6. The generalization ability of the SOM-Hebb classifier depends on the number of neurons assigned to a class [25]. Because of the inferior generalization ability, input gestures must be very close to those used in the training; otherwise, the recognition will fail. In contrast, the recognition accuracy of gesture G7 (a similar gesture to G6) is very high, and it had greater tolerance to changes in the input gesture because G7 was assigned to three neurons. These considerations suggest that the accuracy of G6 could be improved by adding perturbation into the training data [25].

Table 6 lists the performances of various EEG- and ECoG-based recognition systems in the literature. Although a direct comparison cannot be made due to the difference in the number of classes and subjects, it can be said that the recognition accuracy of the proposed system is equivalent to the state of the art. This may be because gestures were defined based on facial movements, which have a large impact on EEG signals.

The whole system was developed on the MATLAB platform. It took about 5.7 s (including a 2 s signal acquisition) for the online recognition system to predict the gesture class, which is slower than real-time processing. In order to apply the facial gesture recognition to robot control, real-time recognition is necessary. We speculate that the recognition speed could be improved if the system were developed using a compiler-based programming language (such as C++).

## 5. Conclusions

In this paper, we proposed an EEG-based facial gesture recognition method that classifies feature vector of a gesture. The feature vector comprises α, β, and θ power bands of the EEG signal. The SOM-Hebb classifier was utilized to classify the feature vectors, and facial gestures were defined by combining facial movements that are easy to detect in EEG signals. Using the proposed method, we developed an online facial gesture recognition system and measured its performance through experiments. The results showed that the accuracy of the system was 98.0% for recognizing two gestures, but this accuracy decreased slightly as the number of gesture classes increased. Notably, when the number of gestures was increased to 7, the accuracy decreased to 83.3%, but this is still high compared to other EEG-based recognition systems.

The implemented online recognition system was developed on the MATLAB platform. It took 5.7 s for the system to complete the recognition flow, including acquisition of the EEG signal, power band conversion, and classification. Although this speed is too slow for real-time operation, improvements in speed can be expected by implementing the entire system in a compiler-based programming language. Improving the speed and developing a real-time recognition system will be addressed in future research. We also plan to improve the recognition accuracy by injecting additive perturbations into the training data. Another issue is the maximum number of gestures with an acceptable recognition rate. In future research, we plan to apply the proposed system to more gestures, such as blinking and breathing, and clarify the relationship between the number of gestures and recognition accuracy.

## Figures and Tables

**Table 1:** Recognition accuracy.

	2 Gestures	4 Gestures	5 Gestures	7 Gestures
Experiment 1	98.00%	92.50%	92.00%	83.33%
Experiment 2	97.14%	96.19%	93.33%	70.47%
Average	97.57%	94.35%	92.67%	76.97%

**Table 2:** Confusion matrix and accuracy of two-class gesture recognition.

	Predicted	
True	G1	G2
G1	50	0
G2	2	48
Accuracy	98.0%	

**Table 3:** Confusion matrix and accuracy of four-class gesture recognition.

	Predicted			
True	G1	G2	G3	G4
G1	28	1	0	1
G2	2	28	0	0
G3	0	2	28	0
G4	0	1	2	27
Accuracy	92.5%			

**Table 4:** Confusion matrix and accuracy of five-class gesture recognition.

	Predicted				
True	G1	G2	G3	G4	G5
G1	28	1	0	1	0
G2	2	28	0	0	0
G3	0	0	30	0	0
G4	0	0	0	30	0
G5	1	1	0	6	22
Accuracy	92.0%				

**Table 5:** Confusion matrix and accuracy of seven-class gesture recognition.

	Predicted						
True	G1	G2	G3	G4	G5	G6	G7
G1	30	0	0	0	0	0	0
G2	0	30	0	0	0	0	0
G3	0	0	30	0	0	0	0
G4	0	0	0	29	0	0	1
G5	0	0	10	8	12	0	0
G6	0	0	14	0	0	15	0
G7	0	0	0	1	0	0	29
Accuracy	83.3%						

**Table 6:** Comparison with other EEG- and ECoG-based recognition systems.

Work	EEG or ECoG	Classifier	Number of Classes	Number of Subjects	Accuracy
[20]	ECoG	LSTM	5 ^†^	7	82.4%
[13]	EEG	SVM	4	3	84.4%
[15]	UHEEG	SVM	2	1	72.7% (moter execution) 71.3% (moter imagery)
[16]	EEG	LSTM	2 ^‡^	109	83.2% (Cross-subject) 98.3% (Intra-subject)
[14]	EEG	HCB	8	5	64.5% (motor imagery)
[19]	ECoG	MLDA	3	2	95.81%
[22]	EEG ECoG	SVM	2	11	77.11% 91.28%
[18]	EEG	RF DT Adaboost SVM	2	2	78.62% 76.20% 72.17% 71.50%
This work	EEG	SOM	2 4 5 7	1	98.0% 92.5% 92.0% 83.3%

UHEEG: ultra-high-density EEG; LSTM: long short-term memory (LSTM); SVM: support vector machine; RF: random forest; HCB: hierarchically combined binary classifier; DT: decision tree; MLDA: multi-class linear discriminant analysis; SOM: self-organizing map; ^†^: Fingerflex dataset; ^‡^: EEG movement dataset.

## Data Availability

Data are contained within the article.
